# Supplementary figures and images for: Impact of bone marrow involvement on outcome in relapsed and refractory transplant eligible diffuse large B-cell lymphoma and transformed indolent lymphoma
Source: PLoS One. 2020 Jul 8;15(7):e0235786. doi: 10.1371/journal.pone.0235786 (PMC7343149; doi:10.1371/journal.pone.0235786)

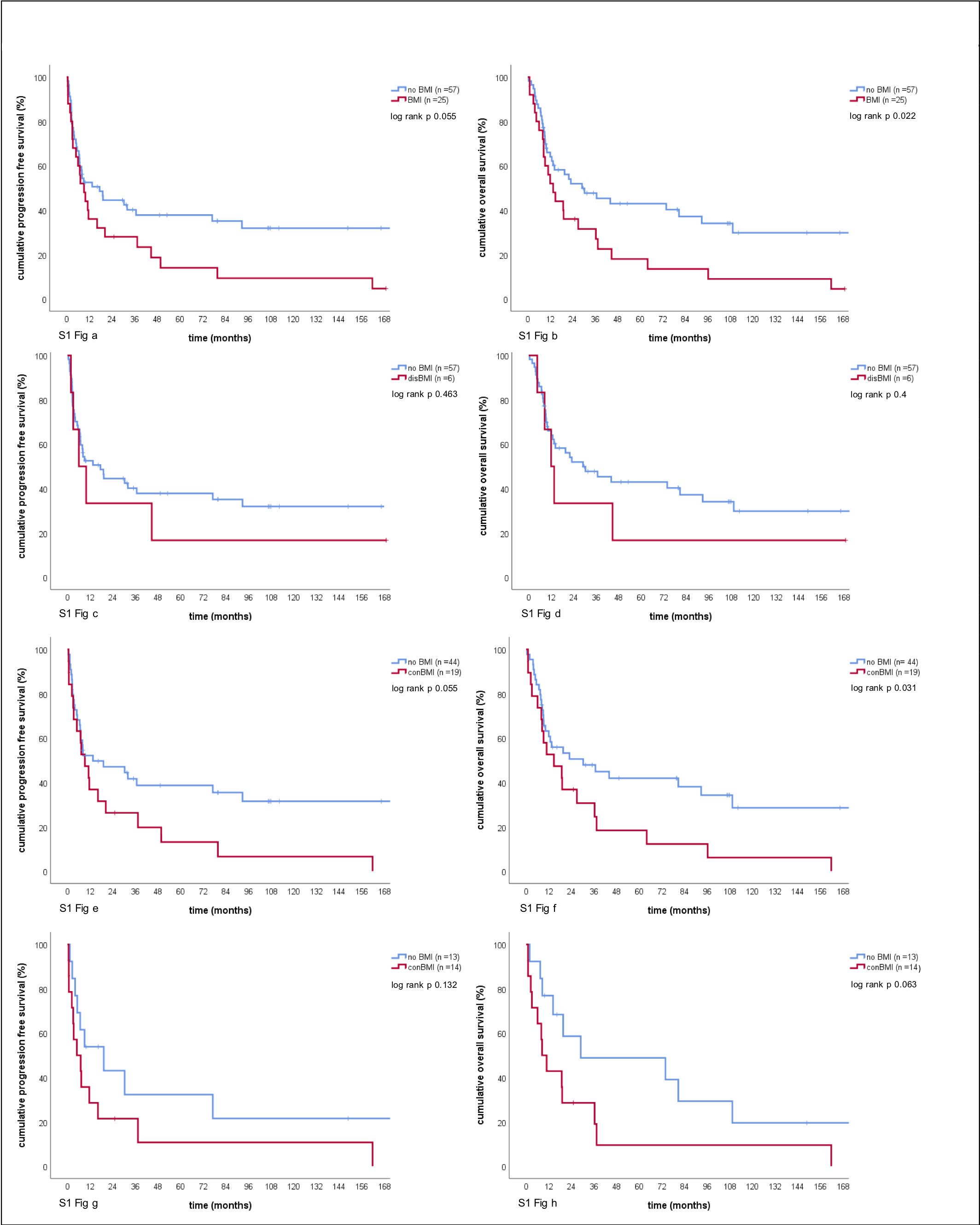

Supplement: S1 Fig — a: KM curve for PFS according to noBMI vs BMI in the total cohort; b: KM curve for OS according to noBMI vs BMI in the total cohort; c: KM curve for PFS according to noBMI vs disBMI in the total cohort; d: KM curve of OS acc ording to noBMI vs disBMI in the total cohort; e: KM curve for PFS according to noBMI vs conBMI in the extensive disease (AA>2) patient subset, f: KM curve for OS according to noBMI vs conBMI in the extensive disease (AA>2) patient subset; g: KM curve for PFS according to noBMI vs conBMI in the nonGCB patient subset; h: KM curve for OS according to noBMI vs conBMI in nonGCB patient subset; Abbreviations: BMI: bone marrow infiltration, noBMI: no bone marrow infiltration, conBMI: concordant bone marrow infiltration, KM: Kaplan-Meier; PFS: progression free survival, OS: overall survival, AA: Ann Arbor, GCB: germinal center B-cell. (TIFF) [file pone.0235786.s001.tiff]
